# On an Improved Mode of Using Refrigeration as an Anæsthetic and as a Remedy

**Published:** 1863-10

**Authors:** Jas. Arnott


On an Improved Mode of using Refrigeration as an Anæsthetic and as a Remedy.—*By Jas. Arnott*, M. D.—It is now, I believe, universally admitted that, by the application of intense cold, pain may be certainly prevented in the numerous operations in which the incision is confined to the skin of the superficial textures; and few will dispute that, in these operations at least, its perfect safety gives it a great advantage over ether or chloroform. But the general opinion is, that it is more troublesome to use than chloroform, and that it is more apt to fail in producing anæsthesia from some oversight or error in the application. This idea has prevented many from employing refrigeration, except in cases where the patients have objected to chloroform, or where there was more than the ordinary risk from its use. In hospital practice, the longer time occupied in effecting congelation has made chloroform to be preferred in almost every case. In consequence of this, many deaths have occurred from the administration of the latter in the most trivial operations—in the extraction of a toe-nail, the opening of an abscess, or the cutting of a wart.

There is nothing singular in this objection, arising from the supposed difficulty or trouble in the use of intense cold. Some of our most valuable remedies have only become generally adopted when the mode of administering them has been simplified. Artificial respiration, galvanism, and several measures resorted to in the treatment of stricture and stone, may be adduced as examples. But the most striking instance of this is found in a therapeutical agent more nearly connected with our present subject. Although sulphuric ether, when properly employed, is not inferior as an anæsthetic to chloroform, the greater trouble attending its administration would have probably very much lessened the use of etherization, had no easier mode of effecting it been discovered. Chloroform is more dangerous than ether, and has on this account been banished from some of the principal hospitals in North America and France, yet so much valued by the great majority of surgeons is its greater ease of administration, that the honor of discovering this means of facilitating anæsthesia, has been almost as keenly contested as that of the great discovery of etherization itself.

Congelation has hitherto been generally produced by placing the freezing materials on the part to be benumbed. In order to ensure success, care must be taken that the ice shall be well pulverized and rapidly mixed with salts constituting the frigorific. The mixture must be applied by means of gauze, or some other thin permeable material; and when the part is not in a horizontal position, a gutta-percha cup fitted to it may be required to keep the frigorific in contact with the skin. Now, all this trouble may generally be avoided by the adoption of an expedient similar to that employed in the therapeutical application of extreme heat. It is rarely the case that a burning substance is applied directly to the part; instead of this, an iron, which has been previously heated in the fire, is used. In a similar way, an iron, or a brass, or a copper implement, of appropriate shape, may be previously cooled in a freezing mixture, and applied with the greatest accuracy to any accessible part, in whatever position this may be. A small, flat, laundry iron, which may be used for pounding the ice, will also answer in a great many cases as the refrigerator. If an extensive or continued refrigeration is required, two such irons, immersed in a semifluid mixture of two or three pounds of ice and salt, may be necessary to replace each other, just as two hot irons are often required for cauterization.

When a metallic body of this description has been cooled to below zero of Fahrenheit, it will often arrest the circulation of the skin the instant it touches it; but more frequently it must be moved and gently pressed on the part for a few seconds, so as to bring a continuous fresh surface in contact with it while the bloodvessels are compressed.

Another expedient, partly resembling that just described, and partly that hitherto in use, consists of a thin metallic bottle (tinned iron or aluminium) completely filled with the frigorific mixture. A Florence flask will sometimes answer the same purpose.

I think the above description is sufficiently minute, and that the surgeon, by the adoption of this method, will no longer have to complain of difficulty or trouble in using congelation, either entirely to prevent pain in minor operations, or to prevent the more acute portion of pain, or that arising from incision of the skin, in operations of a deeper or severer kind. In the preface to his work, entitled "Ten Years' Operative Surgery in the Provinces," Mr. Prichard states, that he "refuses chloroform in the lesser operations wherever ice and salt can be conveniently applied." By means of the metallic refrigerator almost every part will be accessible. The complaint made by Messrs. Perrin and Lallemand in their recent and very complete work on "Surgical Anæsthesia," that congelation has been too much restricted to certain operations, will probably, by this improvement of the process, have no longer any foundation; but that, if I may be allowed to use the words of these writers, "on peut prévoir le moment où, grace a la réfrigeration, l'anesthése pourra être étendue à toute la pratique uselle de la chirurgie." (Page 651.)

It is not, however, in being a safe anæsthetic that the principal value of congelation consists. I am anxious to see it employed as a prompt and certain antiphlogistic in all accessible inflammations. The extraordinary remedial powers of congelation in the various forms of chronic rheumatism, which I have related in former publications, may be attributed partly to its anæsthetic and direct antiphlogistic virtues, and partly to the peculiar counter-irritation which it excites. As promptness of action is eminently characteristic of this remedy, it would be especially serviceable in many of those inflammatory and painful diseases to which soldiers and sailors are liable, and which are at present cured with so much difficulty as to render them long unfit for their duties. Among these may be reckoned sprains and inflammatory affections of the joints, wounds, irritable ulcers, headache, lumbago, and other painful affections, inflammation of various glands, ophthalmia, erysipelas, and other diseases of the skin.

Being convinced, from no little experience, that a short application of intense cold, produced by a frigorific mixture of appropriate strength, constitutes a certain and speedy remedy of every accessible inflammation, as well as a means of preventing pain in operations, without the risk of sudden or (which has been much more frequent) consecutive death attending chloroform, I do not deem that portion of my time misspent which has been employed in devising and describing such a simple and easy mode of making this application as may lead to its general adoption.—*Med. Times and Gaz.*

